# Relationship amongst teratozoospermia, seminal oxidative stress and male infertility

**DOI:** 10.1186/1477-7827-12-45

**Published:** 2014-05-27

**Authors:** Ashok Agarwal, Eva Tvrda, Rakesh Sharma

**Affiliations:** 1 Center for Reproductive Medicine, Glickman Urological and Kidney Institute, Cleveland Clinic, Cleveland, Ohio 44195, USA

**Keywords:** Spermatozoa, Reactive oxygen species, Morphology, Teratozoospermia, Male infertility

## Abstract

**Background:**

Spermatozoa morphology is an important and complex characteristic of the fertilization capacity of male germ cells. Morphological abnormalities have been observed to be accompanied by reactive oxygen species (ROS) overproduction and further damage to spermatozoa, ultimately leading to infertility. Therefore, this study aimed to examine the relationship between seminal ROS production and sperm morphology in infertile teratozoospermic patients as well as in healthy men of proven and unproven fertility.

**Methods:**

Semen samples were collected from 79 patients classified as teratozoospermic and 56 healthy donors (control). Standard semen analysis was performed and spermatozoa morphology was assessed according to the WHO 2010 guidelines. Seminal ROS was measured by chemiluminescence assay. Receiver operating characteristic (ROC) curves were generated, and sensitivity, specificity, cutoff value and area under curve (AUC) were determined.

**Results:**

Sperm morphology was significantly poor in the Teratozoospermic Group compared with the 3 Donor Groups (P < 0.05). Significantly higher levels of ROS (RLU/sec/10^6^ sperm) were seen in the Teratozoospermic group (145.4 (41.5; 555.4) compared to the Donor Groups: All Donors (64.8 (21.1; 198.2), Proven Donors (58.8 (14.2; 79.2) and Proven Donors < 2 years (58.8 (14.2; 79.2) (P < 0.05). ROS correlated negatively with sperm concentration in the All Donor group (r = −0.354; P = 0.021) as well as in the Teratozospermic group (r −0.356; P = 0.002). Using ROC analysis, we established the cutoff values for concentration, morphology and ROS.

**Conclusions:**

The incidence of teratozoospermia may be directly related to the overproduction of seminal ROS. Therefore, besides sperm concentration and motility, spermatozoa morphology should receive an equally important consideration in the overall assessment of male fertility.

## Background

Increasing fertilization incompetence has become a major concern for males and females of reproductive age. As male factor is thought to contribute to approximately 40% of all infertility cases, an accurate semen analysis should be the keystone of the assessment of male fertilization potential [1,2].

Sperm count and sperm motility are typically the first diagnostic markers to be evaluated when studying semen quality. Morphology of the sperm cell is an underrated semen parameter, mainly because of the lack of a commonly accepted evaluation method as well as a general cut-off value [2-4]. Nevertheless, the morphologic characteristics of the sperm cell are the outcome of highly complex cellular modifications occurring during spermatogenesis [3,5,6]. The resulting percentage of abnormal spermatozoa as well as specific structural abnormalities may serve as an indicator of a defective mechanism related to spermatozoa production and/or maturation [1] and is a valuable predictor of spontaneous pregnancies and fertilization success in assisted reproductive technology (ART) [7-9]. Furthermore, abnormal spermatozoa morphology has been linked not only to a decrease of traditional parameters of semen quality [10-12] but also to an increase in contemporary markers of sperm damage, such as DNA fragmentation [1,2] or reactive oxygen species (ROS) overproduction [13,14].

A common origin of both pathological spermatozoa and ROS may be found within the sperm membrane remodeling during spermatogenesis. Due to failures in the process, such as abnormal head-tail attachments, incomplete acrosomal development or alterations in the sperm cytoskeleton [15], spermatozoa exhibit cytoplasmic residues leading to the creation of both morphologically abnormal structures as well ROS [16]. Pathological spermatozoa, together with leukocytes, are considered to be the primary source of free radicals in semen [14,17,18]. Additionally, ROS overproduction has been linked to oxidative damage of the poorly protected sperm cell. The polyunsaturated fatty acids (PUFA), present in large quantities in the cytoplasmic membrane of spermatozoa, are the primary target for deleterious peroxidation, which leads to a decreased membrane fluidity and further structural defects of the sperm cell [19,20].

Although several studies have highlighted an association between spermatozoa morphology and ROS present in semen [16,21,22], the exact mechanism linking both the abnormal sperm morphological forms and the oxidative balance within the sperm cell have not been investigated. Therefore, in this study, we examined the relationship between seminal ROS production and sperm morphology in infertile teratozoospermic patients as well as in healthy men of proven and unproven fertility. The results may confirm the complex biological relationship between teratozoospermia and oxidative stress and possibly help to explain the causes of male infertility in some men.

## Methods

Following approval from the Cleveland Clinic Institutional Review Board (IRB), 135 subjects were enrolled in the study: 56 healthy male volunteers and 79 patients diagnosed with teratozoospermia (percentage of spermatozoa with < 4% normal morphology).

The inclusion criteria for the infertile patients were as follows: all subjects attended the male infertility clinic for fertility issues. All of these men were evaluated for proven male-factor infertility as assessed the male infertility specialist. All of them underwent history, physical and laboratory evaluation. None of them had female factor infertility in their partners. Our exclusion criteria were: azoospermia, incomplete semen analysis results or inadequate semen sample for ROS measurement.

The donors were healthy males, 20 – 35 years old whose semen samples fulfilled the criteria established by the WHO 2010 guidelines for semen analysis i.e. normal semen parameters [23]. Of the 56 donors, 28/56 (50%) were of proven fertility (having established a successful pregnancy in the past), and 16/56 (28.6%) had initiated a pregnancy in the past 2 years.

The inclusion criteria for the All Donor group were: 1) normal semen parameters; 2) no sexually transmitted infections; 3) no recreational drug use, and 4) may or may not have initiated a pregnancy in the past. The Proven Donor group (n = 28) included men who had initiated a pregnancy at some point. The inclusion criteria were: 1) normal semen parameters; 2) no sexually transmitted infections; 3) no recreational drug use and 4) Initiated a pregnancy in the past. The third donor group (Proven Donors < 2 years) (n = 12) included men who had initiated a pregnancy in the past two years. The Inclusion criteria were: 1) normal semen parameters; 2) no sexually transmitted infections; 3) no recreational drug use and 4) initiated a pregnancy within the past 2 years. The exclusion criteria for the donors were the following: azoospermia, incomplete semen analysis results or inadequate semen sample for measurement of ROS.

### Semen analysis

Semen samples were collected by masturbation after 2–3 days of sexual abstinence. After liquefaction, a complete semen analysis was performed to evaluate the sperm parameters according to the World Health Organization (WHO) guidelines [23]. Sperm concentration and percentage motility analysis were done using a MicroCell counting chamber (Vitrolife, San Diego, California).

### Measurement of white blood cells

The presence of peroxidase positive leukocytes (neutrophils and macrophages) in semen was assessed by a myeloperoxidase- staining test. 20 μL of liquefied semen specimen was mixed well with 20 μL of phosphate-buffered saline (PBS) (pH 7.0) and 40 μL of benzidine solution. The mixture was allowed to sit at room temperature for 5 minutes. Peroxidase positive leukocytes that stained brown were counted by a Makler counting chamber (Sefi Medical, Haifa, Israel) under a bright-field objective (magnification, × 20). The results after correction for dilution were recorded as × 10^6^ peroxidase-positive leukocytes/mL of semen. A seminal leukocyte concentration of ≤1 × 10^6^WBC/mL was considered normal [23].

### Assessment of sperm morphology

Thin smears of the well-mixed ejaculated semen were prepared in duplicate by placing 2–5 μL (depending on the sperm concentration) on clean slides. After air drying, the slides were stained using Diff-Quik kit (Baxter Healthcare Corporation, Inc., McGaw Park, IL) and graded on the basis of the Kruger’s Strict criteria and cutoff value established by WHO 2010 guideline [23]. A total of 100 spermatozoa were scored per slide using bright field illumination and an oil immersion objective with a total magnification of × 2000. At least ten high-power fields selected at random from different areas of the slide were examined.

### Measurement of reactive oxygen species

ROS levels in seminal ejaculates were measured by chemiluminescence assay using luminol (5-amino-2, 3- dihydro-1, 4-phthalazinedione; Sigma, St Louis, MO) as the probe. The test samples consisted of luminol (10 μL, 5 mM) and 400 μL of semen. Negative controls were prepared by replacing the sperm suspension with 400 μL phosphate buffered saline. Positive control included 400 μL of PBS and 50 μL of hydrogen peroxide (30%; 8.8 M) in triplicates. Chemiluminescence was measured for 15 min using a Berthold luminometer (Autolumat Plus 953, Oakridge, TN). The results were expressed as relative light units (RLU)/sec/10^6^ sperm [24].

### Statistical analysis

The data were analyzed using inbuilt functions within the Statistical Package for Social Science (SPSS UK Ltd., Chertsey, Surrey, UK). Summary statistics are presented as mean and standard deviation (SD). Univariate comparison of continuous variables among the groups was performed with the Kruskal-Wallis test. Simultaneous multiple pairwise comparisons among groups were performed with the Conover–Inman test, which is simply Fisher’s least significance difference method performed on ranks. Spearman’s rank correlation test was used to provide a distribution-free test of independence between sperm ROS production and sperm attributes. All hypothesis testing was two-tailed; P < 0.05 was considered statistically significant. Forward stepwise logistic regression analysis was used to identify a suitable model predicting high sperm ROS production.

## Results

### Semen parameters

Tables 1, 2 and 3 represent the sperm parameters in three healthy donor groups (unproven fertility, any proven fertility, and proven fertility within the previous 2 years) as compared to the teratozoospermic patients. While the spermatozoa concentration was not significantly different between the patients and the donors, a significant (P < 0.05) decrease in the seminal volume was observed in the Teratozoospermic Patients (3.27 ± 1.62 mL) as compared with the Proven Donors (4.24 ± 2.13 mL) and Proven Donors < 2 years (5.03 ± 2.22 mL). Spermatozoa motility was higher in the Teratozoospermic group (57.66 ± 12.33%) compared with both Proven Donors (50.85 ± 13.52%) and Proven Donors < 2 years (49.88 ± 8.68%).

**Table 1:** Comparison of semen parameters (mean ± SD) in All Donors (n = 56) and Teratozoospermic Patients (n = 79)

**Parameter**	**All donors**	**Teratozoospermic patients**	**Sensitivity (%)**	**Specificity (%)**	**Cutoff value**	**AUC value**
**Volume (mL)**	3.36 ± 2.02	3.27 ± 1.62	89.7	23.2	5.0	0.482
**Concentration (× 10**^**6**^**/mL)**	54.26 ± 32.19	60.64 ± 53.58	55.7	63.6	41.7	0.529
**Motility (%)**	53.70 ± 15.00	57.66 ± 12.33	25.3	77.8	45.5	0.400
**Normal morphology (%)**	6.93 ± 3.91	1.52 ± 1.12*	100	78	3.5	0.916
**Endtz test (WBC/mL)**	1.04 ± 2.54	0.25 ± 0.87	N/A	N/A	N/A	N/A
**ROS (RLU/sec/10**^**6 **^**sperm)**	64.8 (21.1; 198.2)**	145.4 (41.5; 555.4)*; **	63.9	65.1	85.9	0.614

*Significant if P < 0.05; **Values are represented as median (25^th^, 75^th^ percentile).

**Table 2:** Comparison of semen parameters (mean ± SD) in Proven Donors (n = 28) and Teratozoospermic Patients (n = 79)

**Parameter**	**Proven donors**	**Teratozoospermic patients**	**Sensitivity (%)**	**Specificity (%)**	**Cutoff value**	**AUC**
**Volume (mL)**	4.24 ± 2.13	3.27 ± 1.62*	89.7	35.7	5.0	0.633
**Concentration (× 10**^**6**^**/mL)**	60.07 ± 33.44	60.64 ± 53.58	55.7	71.4	41.7	0.578
**Motility (%)**	50.85 ± 13.52	57.66 ± 12.33*	-	-	-	-
**Normal morphology (%)**	7.00 ± 4.35	1.52 ± 1.12*	100	73.7	3.5	0.913
**Endtz test (WBC/mL)**	0.00 ± 0.00	0.25 ± 0.87	N/A	N/A	N/A	N/A
**ROS (RLU/sec/10**^**6 **^**sperm)**	75.8 (33.3; 147.8)**	145.4 (41.5; 555.4)*; **	61.1	70.4	95.3	0.638

*Significant if P < 0.05; **Values represented as median (25^th^, 75^th^ percentile).

**Table 3:** Comparison of semen parameters (mean ± SD) in Proven Donors < 2 years (n = 16) and Teratozoospermic Patients (n = 79)

**Parameter**	**Proven donors <2 y**	**Teratozoospermic patients**	**Sensitivity (%)**	**Specificity (%)**	**Cutoff value**	**AUC value**
**Volume (mL)**	5.03 ± 2.22	3.27 ± 1.62*	89.7	50	5.0	0.746
**Concentration (× 10**^**6**^**/mL)**	61.59 ± 23.93	60.64 ± 53.58	55.7	87.5	41.7	0.652
**Motility (%)**	49.88 ± 8.68	57.66 ± 12.33*	27.8	75	46.5	0.33
**Normal morphology (%)**	6.77 ± 4.95	1.52 ± 1.12*	100	69.2	3.5	0.883
**Endtz test (WBC/mL)**	0.00 ± 0.00	0.25 ± 0.87	N/A	N/A	N/A	N/A
**ROS (RLU/sec/10**^**6 **^**sperm)**	58.8 (14.2; 79.2)**	145.4 (41.5; 555.4)*; **	61.1	93.8	95.3	0.73

*Significant if P < 0.05; **Values are represented as median (25^th^, 75^th^ percentile).

The highest percentage of morphologically normal spermatozoa was seen in Proven Donors followed by All Donors (Tables 1, 2, 3). Significantly lower percentages of morphologically normal spermatozoa were seen in the Teratozoospermic group (1.52 ± 1.12%) when compared to all three Donor groups (P < 0.05) (Tables 1, 2, 3).

Sperm morphology was positively correlated with concentration in Donor groups (P = 0.009; P = 0.037 and P = 004, respectively). Similarly, normal morphology was correlated with motility in All Donors (P = 0.001) and Proven Donors (P = 0.032). Furthermore, a significant correlation was found between spermatozoa morphology and semen volume in Teratozoospermic Patients (P = 0.044) (Table 4).

**Table 4:** Correlations between the semen quality parameters, spermatozoa morphology and ROS production in the Donor and Teratozoospermia groups

**Experimental group**	**Parameter (1)**	**Parameter (2)**	**n**	**Spearman correlation**	**P-value**
**All Donors**	Normal morphology	Concentration	56	0.407	0.009
	Normal morphology	Motile sperm	56	0.508	0.001
	ROS	Concentration	56	−0.354	0.021
**Proven Donors**	Normal morphology	Concentration	28	0.481	0.037
	Normal morphology	Motile sperm	28	0.494	0.032
**Proven Donors < 2 y**	Normal morphology	Concentration	16	0.733	0.004
**Teratozoospermic Patients**	Motility	Concentration	79	0.346	0.002
**Teratozoospermic Patients**	Normal morphology	Volume	79	0.228	0.044
**Teratozoospermic Patients**	ROS	Concentration	79	−0.356	0.002

The sensitivity, specificity, cutoff value and area under curve (AUC) for the three donor groups and teratozoospermic patients are shown in Tables 1, 2, 3. Sperm morphology showed high specificity and AUC. The cutoff values for concentration were similar in the 3 Donor groups (41.7, 41.75 and 41.75 × 10^6^/mL). Morphology also had a similar cutoff value of 3.5% in the 3 Donor groups and Teratozoospermic group.

### ROS production

**Figure 1:**
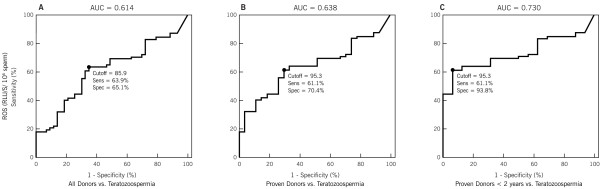
**Receiver operator characteristic (ROC) curves for ROS in (A) All Donors; (B) Proven Donors and (C) Proven Donors < 2 years fertility vs. Teratozoospermia group.** ROC curves shows the area under the curve for ROS production in Patients with teratozoospermia when compared to All Donors, Proven Donors, and Proven Donors who had initiated pregnancy in the last two years.

All the Donor groups were characterized by normal ROS levels, as shown in Tables 1, 2, 3 as well as Figure 1. The lowest ROS values (median (25^th^, 75^th^ percentile) were detected in proven donors < 2 years (58.8 (14.2; 79.2) RLU/sec/10^6^ sperm). The highest ROS production was recorded in the Teratozoospermic patients group (145.4 (41.5; 555.4) RLU/sec/10^6^ sperm), (P < 0.05) in comparison with all donor groups. Furthermore the ROS production was negatively correlated with the spermatozoa concentration in All Donors (r = −0.354; P = 0.021) as well as Teratozoospermic Patients (r = −0.356; P = 0.002; Table 4).

The sensitivity, specificity, cutoff value and area under curve (AUC) for ROS in the 3 Donor groups and Teratozoospermic Patients is shown in Tables 1, 2, 3 and Figure 1. ROS showed high specificity and AUC in the Proven Donors < 2 years and Teratozoospermic group. Sensitivity was comparable in all the groups. The cutoff value was 85.9, 95.3 and 95.3 RLU/sec/10^6^ sperm in the 3 Donor groups compared with Teratozoospermic group (Figure 1).

## Discussion

Of male patients referred for fertility evaluation, 25-50% are diagnosed with idiopathic infertility [5,6,25]. Therefore, an accurate semen analysis plays a crucial role in the management of infertile couples and treatment options.

Ideally, the three traditional markers of semen quality (sperm count, motility, morphology) should be strongly interrelated to reflect their contribution to a successful fertilization. However, it is known now, that even if the sperm concentration or motility is good, a morphological defect may be the single most important factor reflecting the actual fertilization capacity of the sperm. (Tables 1, 2, 3) [6,10,18,20]. While positive correlations have been found mainly in *in vivo* studies, negative associations and the independent character of morphology has been also been demonstrated with fertilization success. Therefore, it is necessary to point out both – the status of morphology within the traditional semen parameters as well as its status as an individual marker. [4,25-27].

The patient population in this study presented with good sperm count and motility but poor morphology, relating to previous observations that this parameter may reflect best the actual ability of the sperm cell to successfully fertilize the oocyte [28]. In fact, strict morphology has become a significant prognostic value in assisted reproduction, as in the case of intrauterine insemination [29], *in vitro* fertilization (IVF) [9,30] and intracytoplasmic sperm injection (ICSI) [7,31]. Regardless of the assisted reproductive technique selected, using spermatozoa with morphological abnormalities leads to lower fertilization and pregnancy rates, as well as a higher risk of fetal DNA damage [28-31].

As traditional markers of semen quality have been defined and studied on numerous occasions, attention is driven towards new and alternative diagnostic tools, such as the evaluation of free radical production, providing explanations to the gaps between semen quality and the actual fertilization potential [13,14,16,21]. Our results show significant differences in the ROS levels between the Teratozoospermia group and all the Donor groups (Tables 1, 2, 3).

Overproduction of ROS and oxidative damage to the sperm cell has been acknowledged as one of the leading causes and/or secondary complications connected to the decreasing fertility potential in males [32]. Low levels of ROS (physiological levels) are needed to promote essential signaling pathways to promote spermatozoa maturation, capacitation, hyperactivation and acrosome reaction [33].

Excessive levels of ROS in the male reproductive system may be generated by two sources: immature and/or pathological spermatozoa and activated leukocytes. Leukocytes are known to generate significantly larger levels of ROS. Immature and/or pathologic spermatozoa in males with sperm abnormalities are expected to make a greater contribution to ROS in teratozoospermic than in normospermic males, as reflected by Gil-Guzman et al. [34] and Oborna et al. [18].

We evaluated the leukocyte concentration using the peroxidase or the Endtz test. We did not separate leukocytes from the seminal ejaculates when performing the ROS measurement. The Endtz test result in our study shows that the concentration of peroxidase-positive cells in teratozoospermic subjects was very low and non-significant when compared to the Donor Group. In fact, the concentration of 0.25 ± 0.87 × 10^6^ wbc/mL was lower than in the All Donor group (1.04 ± 2.54 wbs/mL) although not significant and furthermore coupled with the WHO threshold of 1.0 × 10^6^ wbc/mL. We did not classify sperm abnormalities into head, mid-piece and tail abnormalities according to the WHO 1999 criteria. However, based on this observation, we assume that the ROS overproduction in the Patient Group was related primarily to the high occurrence of spermatozoa malformations. Moreover, none of the patients had elevated levels of white blood cells in the ejaculate and there the source of ROS was largely a product of increased ROS from the spermatozoa. High ROS production in the absence of leukocytes especially the granulocytes indicates the source of high ROS to be morphologically abnormal spermatozoa. This may also clarify the hypothesis that the cytoplasmic membrane could be the primary structure to be involved in morphological abnormalities of the spermatozoa. At the same time, it is the main sperm structure to be attacked by ROS [35].

Our data may supplement previous reports showing association between defective sperm function and excess cytoplasmic enzymes such as superoxide dismutase, lactic acid dehydrogenase and creatine kinase and glucose-6-phosphate dehydrogenase [21,33,36-39]. These are directly involved in the oxidative balance of spermatozoa with the latter being highly interrelated with peroxidative damage to the sperm cell [40,41]. Furthermore, Ghani et al. [42] showed a significantly elevated expression of NOX5, a novel NADPH-oxidase and prime candidate for the ROS production in the acrosomal, equatorial, post-acrosomal regions of abnormal spermatozoa. Moreover, a significant positive correlation was observed between the NOX5 activity and the frequency of sperm with abnormal morphology.

Interestingly, no significant correlation was found between ROS production and spermatozoa motility, an observation contrary to a number of studies performed on ejaculates from healthy males as well as infertile patients [17,18,21,22]. On the other hand, Whittington et al. [43] as well as Desai et al. [44] found no correlation between the motility parameters and ROS assuming that similarly to the spermatozoa morphology, seminal ROS might be an independent marker of fertility in clinical settings. Moreover, significant connections between spermatozoa morphology and ROS were recorded in these studies proving strong interrelations between the two parameters, similar to our results (Table 4).

This study has enabled us to define the cutoff values as well as the sensitivity, specificity and the area under curve (AUC) for a variety of sperm characteristics, including morphology as well as for ROS comparing the Donor and Teratozoospermic groups. This information is important as the cutoff values may be used when identifying the different patients and donors in the future. Furthermore, we have established the cutoff values for ROS in the general donor and patient population but not specifically comparing concrete subsets of donors with unproven and proven fertility and a subset of infertile men exhibiting teratozoospermia.

Several earlier studies [45-49] have used the ROC curves to demonstrate the importance in establishing the cutoff and threshold values for different semen parameters based on clinical, rather than empiric data. Using ROC curve analysis, Ombelet et al. [45] showed that the sperm morphology was the best semen parameter with the highest prediction power (AUC = 78%) and a cutoff value of 10%. A similar threshold value was detected by Günalp et al. [46] with an AUC of 69.7%. On the contrary, and similar to our results, Menkveld et al. [47] found a much lower cutoff value for morphology (4%), but with a good predictive value based on an AUC of 78.2%.

Moreover, Guzick et al. [48], using the CART analysis, was able to establish a cutoff value for spermatozoa morphology in fertile (>12%) as well as in subfertile subjects (<9%). Their ROC curve analysis showed that morphology had the best predictive power based on an AUC of 66%.

The ROC analysis for the ROS production has been performed in a few studies only. According to Allamaneni et al. [49], the optimum ROS cutoff value to identify patients with oxidative stress in neat semen was 0.185 × 10^6^ cpm/20 × 10^6^ sperm, with an AUC value from 0.57– 0.80. Furthermore, in the study by Desai et al. [50] the calculated ROS cutoff value to differentiate between fertile and infertile subjects was 0.0185 × 10^6^ cpm/20 × 10^6^ sperm, with the unadjusted positive predictive value of 82.4% and the negative predictive value of 77.8%. Similarly, we have established the ROS cutoff value in the seminal ejaculate of 93 RLU/sec/10^6^ sperm with a specificity of 70.4% and sensitivity of 61.4% and area under curve of 68% [24,51]. Our AUC values varied between 61.4% and 73%, proving conclusions from both manuscripts [24,51] that the ROS measurement has an important clinical relevance as a test used for infertility screening.

## Conclusions

Based on the compatibility of results from our study along with previous observations, we strongly support the hypothesis that there is a direct relationship between spermatozoa morphology and oxidative balance. Disturbances in spermatozoa production and maturation may have a dramatic impact on the structural characteristics as well as free radical production in semen. Furthermore, we emphasize that spermatozoa morphology is probably the most relevant parameter of traditional semen evaluation, providing information of the fertilization potential, which in combination with modern markers of semen quality, such as ROS production, may have the best indication value of poor semen quality in the laboratory assessment of infertile men. At the same time, we suggest further comparative studies connecting the spermatozoa morphology and ROS production with further markers of semen quality, such as DNA integrity or seminal antioxidant status.

## Competing interests

All authors declare no competing financial interests.

## Authors’ contributions

AA conceived the idea, supervised the study, and edited the article for submission. ET conducted the study and helped with the writing of manuscript, and preparation for submission. RKS helped with the review and editing of the manuscript. All authors read and approved the final manuscript.
